# A Case of Richter's Strangulated Hernia

**Published:** 1914-09

**Authors:** C. A. Moore

**Affiliations:** Assistant Surgeon, Bristol General Hospital


### A CASE OF RICHTER'S STRANGULATED HERNIA.

BY

C. A. Moore, M.S.Lond., Ch.M.Bristol, F.R.C.S.,

*Assistant Surgeon, Bristol General Hospital.*

---

The condition known as Richter's hernia is one not very commonly met with, but it is especially serious owing to the delay which often occurs in establishing the diagnosis, the result of the vagueness of the symptoms which is not infrequently present.

In a case of this nature the tumour may be so small as to escape detection, although pain, tenderness, or resistance may be felt. Constipation is not always absolute, some flatus or even fæces passing; this for the obvious reason that the lumen of the gut is not totally mechanically obstructed, nor is there necessarily so great a degree of vascular occlusion as to cause a state of dynamic obstruction. Vomiting is not always a prominent feature, and rarely becomes stercoraceous. One or more intermissions in the course of the disease often occur, a point to which attention was called by Sir William Bennett in his *Lectures on Hernia*. There was one distinct intermission in the case described below, in which also the uncertainty of the diagnosis was considerable.

W. H., æt. 29, was seen on the ninth day of his illness. He said he had had a "rupture," operated on by the writer a year previously, and this statement seemed to be confirmed by the presence of a scar in the right inguinal region. Reference to my notes later, however, proved that the operation was for a cyst of the epididymis. Apart from that, so far as he knew, he had never suffered from rupture, nor from any previous attack similar to the one under notice.

This commenced without apparent cause, eight days before the patient was seen, with severe griping pains across the epigastrium and frequent vomiting. The symptoms abated after two days, but then recurred, and had continued off and on ever since. The pain was very little in the lower abdomen, and none was complained of in the groin.

The patient looked extremely ill, with a typical "abdominal" facies, sunken eyes and dilated pupils, and was rolling about in obviously severe pain. The pulse rate was 88 per minute, and the temperature 99.8°.

In the region of the right femoral ring was a very small, firm nodule, quite free from any tenderness. It was so small that it might well have been a gland, and too small to hope to be able to elicit any of the classical signs of strangulated hernia. The abdominal signs were puzzling. There was no distension, nor were any distended coils of intestine visible. In the epigastrium, where the complaint of pain was most severe, the abdomen was quite board-like in its rigidity. It was rigid in the lower part, but distinctly less so than above, and more on the right side than on the left. Examination for shifting dulness was omitted owing to the patient's cries and restlessness. Liver dulness was present.

What, then, was the diagnosis? Had he a small strangulated femoral hernia of eight days' standing? If so, why the severe epigastric pain and rigidity? Had he a perforated gastric or duodenal ulcer, or was his condition due to an inflamed appendix, or possibly acute pancreatitis?

On the whole, in view of the uncertainty, it seemed best to explore the femoral swelling to exclude strangulated hernia.

An incision was made in this situation, and with some difficulty a minute sac was found. On opening this, the contents proved to be a small viscus, red in colour, not gangrenous, and looking exactly like the tip of an inflamed appendix. It could not be drawn down by gentle traction, so Gimbernat's ligament was nicked : the contents of the sac then promptly disappeared into the abdomen.

It seemed possible that the condition, after all, was one of appendicitis, but in a hernial sac, and in view of the serious abdominal symptoms it seemed advisable to investigate further.

Accordingly, the abdomen was opened through the margin of the right rectus muscle. The peritoneum contained almost clear fluid in considerable quantity, with numerous flakes of lymph. A coil of bowel immediately presented, leaking fæces freely.

What had happened was this. The small object in the sac was a diverticulum, scarcely larger than a good-sized pea, and apparently artificial, on the convexity of a coil of small intestine. Traction on this had so sharply kinked the bowel as to cause ulceration at the kink on the concavity. The ulcer had given way and infected the peritoneal cavity.

The bowel itself was somewhat œdematous, but very little distended, and otherwise in good condition. The hole was too large to permit of closure without danger of obstructing the lumen of the bowel, even granted that stitches could be relied on to hold in the damaged tissues. The bowel containing the fistula was excised with about four inches on either side, and continuity restored by end-to-end anastomosis. The abdomen was closed and drained through a tube in the femoral canal.

The operation was followed by no marked shock, but paralysis of the bowel caused a highly critical condition for the next few days. Enemata were useless, but a course of intramuscular injections of pituitary extract given simultaneously with a turpentine enema produced the desired result. After the bowels were open the patient's condition rapidly improved. Both wounds suppurated mildly from the fæcal contamination, but rapidly healed up, and the man was up in three weeks.

Incidentally it may be remarked that in the writer's opinion this case adds one more to the long list of those whose lives have been saved by the power of pituitary extract to cause contraction of involuntary muscle fibre, and to dispel that fatal complication of intestinal paralysis following operations for obstruction or peritonitis.

Cases of hernia—strangulated or otherwise—in which the whole circumference of the gut is not involved have been described, for a very long time. Attention was apparently first called to them in an essay by Lavater, published in 1672. In 1714 Littré noticed cases in which a diverticulum from the bowel was involved in a hernia. He discussed the possibility of such diverticula being of congenital origin, but dismissed the idea as too improbable. Morgagni, in 1741, published a case in which strangulation of only part of the circumference of the bowel caused death. Richter's work was published in 1799, and in common with other early workers on the subject he recognised two classes of case, according to whether an actual diverticulum was or was not present, but he made no other distinction between them. Except that Meckel demonstrated beyond doubt that the diverticulum which now bears his name was, of congenital origin, the subject received no special attention for nearly a century. Treves, in a paper read before the Medico-Chirurgical Society in 1887, gave Littré's name to the cases with a diverticulum, and Richter's to those in which a part of the bowel-wall was involved. This nomenclature is now generally adopted, and at least keeps alive the names of two early workers on the subject, even if the two names do seem to have been attached to the respective classes of case somewhat empirically.

I have called the above case Richter's hernia, since the diverticulum appeared to be the result of traction, and not of the nature of a Meckel's diverticulum. I did not observe how far from the ileo-cæcal valve the diverticulum was situated.

